# The Operative Treatment of Acute Epididymo-Orchitis

**Published:** 1912-04-06

**Authors:** 


### THE OPERATIVE TREATMENT OF ACUTE EPIDIDYMO-ORCHITIS.

THE stereotyped treatment for the above affection in the past has for many years been strictly palliative. The patient has been put to bed on a light non-stimulating diet from which alcohol in every form has been rigidly excluded. He has been treated locally with lead lotion, strapping, very occasionally leeches, and constitutionally by salines and antimony. Under this regimen the vast majority of cases got well, or apparently well. Nevertheless, as our knowledge of the pathology of the condition increased, it was found that not infrequently under this treatment abscesses appeared from which the gonococcus could be isolated in pure culture; cases apparently well proved years later on marriage to be still infectious; and not infrequently when the condition was bilateral, or a subsequent infection of the other testis occurred, sterility was the result.

It is not surprising, therefore, to find that in recent years there has been a steadily increasing tendency towards surgical interference in this condition. The silence of text-books on the subject, however, is almost complete, and this is all the more remarkable when on looking into the literature on the subject, we find how long established the suggestion of operative treatment in reality is, for as early as 1864 Henry Smith, a surgeon at King's College, published an account of some twenty cases of "orchitis," as he called it, treated by incision.

### AN ACCIDENTAL SUCCESS.

The story of his first case is not uninteresting, and may be referred to in some detail. He was led to the operation by a mistake in diagnosis. In July 1863 a young man presented himself with what was diagnosed as an abscess of the testicle. On opening into this, however, with a sharp bistuary no pus was found, only a little serum and blood exuded, and the tubes of the testis herniated through the incision. Deeply chagrined, the surgeon strapped the testis, and dismissed the patient. Two days later, however, to his intense surprise, the man returned wonderfully better, the pain, swelling, and inflammation gone, and no testicular substance now showing. Thinking the matter over, Smith came to the conclusion, therefore, that "the quantity of blood and serum abstracted was so small that the cessation of pain and diminution in swelling could hardly be due to this cause, but that the free division of the fibrous coat enveloping the testis, and the consequent removal of tension, was the secret of the success." When he published * his paper, with the results of twenty cases, a year later he found, however, that his discovery had already been anticipated, and that the line of treatment advocated was that usually adopted at the Hôpital de Midi in Paris.

The treatment would, however, appear to have fallen into desuetude, for nothing further is heard of it in English medical literature. Probably in those pre-Listerian days cases of accidental infection caused results that threw the operation into disrepute. In France, however, it would seem never to have quite died out. Vidal (de Casis) carried a bistuary into the testicular substance, and opened the tunica albuginea by several incisions, in certain cases of parenchymatous orchitis; and Velpeau punctured the tunica vaginalis in epididymo-orchitis in every case with symptoms of hydrocele, noticing that the greater the fluid distension the greater the pain. Monod and Terillon also operated for the condition. In 1892 Trekaki re-introduced the subject,† recording the satisfactory results obtained in twenty cases treated by semicircular incision of 2 to 3 cm. through the tunica vaginalis. Later Baermann,‡ on investigating the pathology of the condition, found that suppuration was not uncommonly associated with hydrocele, and that puncture relieved pain, caused a more rapid cure, and saved the canal from occlusion.

### DR. HAGNER'S PROCEDURE.

The most recent method of operating is that of Dr. Hagner, of Washington. He makes an incision 6 to 10 cm. long vertically on the outer side of the testis, over its junction with the epididymis. The tunica vaginalis is opened, and the hydrocele evacuated. The organ is next turned out through the opening, and multiple puncture is performed with a fine tenotome through the fibrous coat of the epididymis, especially where induration is felt. If pus is found the cavity is evacuated. Finally, after replacing the testis the incision is sewn up with a continuous suture. Hagner operates only on about ten per cent. of his cases, and those operated on now amount to thirty-three. Among these pus was found present in twenty-five, and suppuration was four times as frequent in the globus minor as in the globus major. In thirty-one cases there was an accompanying hydrocele, in one other the walls of the tunica were adherent with plastic lymph. The results obtained were: rapid relief of pain, and a fall of temperature and pulse to normal inside thirty-six hours. In one case the leucocytosis fell from 33,000 to 8,400 in forty-eight hours. The average time in bed was five days. All these cases, it must be noted, were operated on because of the severity of the symptoms.

Dr. G. G. Smith, summing up the evidence § after treating five cases by Hagner's method, came to the conclusion that the ordinary cases of epididymo-orchitis kept in bed with tight bandaging for three to five days do equally well with those operated on, but in severe cases, with excruciating pain, marked toxæmia, and symptoms of collapse, and in recurring cases, operation is indicated. To these should probably be added cases of bilateral infection, as the more rapid the cure the less chance there is of occlusion of the vas, and its sequel—sterility.

* *Lancet*, August 6, 1864, p. 149.
† *Gazette Médicale de Paris*, April 16, 1892, p. 185.
‡ *Deutsche Med. Woch.*, October 1, 1903, p. 720.
§ *Boston Med. and Surg. Journal*, February 2.

